# Naval sonar induces an anaerobic swimming gait in beaked whales

**DOI:** 10.1038/s41598-025-22490-5

**Published:** 2025-11-05

**Authors:** L. M. Martín López, S. Isojunno, D. Cade, K. Colson, I. Paradinas, P. J. O. Miller, A. Fahlman, L. S. Hickmott, F. Visser

**Affiliations:** 1 SMRU, Scottish Oceans Institute, University of St Andrews, St Andrews, Fife, KY16 8LB UK; 2 Asociación IPAR Perspective, 33508 El Allende Llanes, Asturias Spain; 3 Hopkins Marine Station, Stanford University, 93950 California, USA; 4 Kelp Marine Research, 1624 CJ Hoorn, The Netherlands; 5 AZTI, Txatxarramendi Ugartea z/g, 48395 Sukarrieta, Bizkaia Spain; 6 Fundación Oceanogràfic, Eduardo Primo Yúfera (Científic), 1B, 46013 Valencia, Spain; 7 Global Diving Research SL, CALLE FELIX PIZCUETA, 5 - PT9, Sanlucar de Barrameda, 46004 Valencia Spain; 8 IFM, Linköping University, Linköping, 581 83 Sweden; 9 Open Ocean Consulting, Hampshire, GU32 2EY UK; 10 Royal Netherlands Institute for Sea Research, PO Box 59, 1790 AB Den Burg, The Netherlands

**Keywords:** Beaked whales, Anthropogenic noise, Behavioural response, Naval sonar, Diving physiology, Marine mammals, Behavioural ecology, Marine biology

## Abstract

Naval sonar can disrupt beaked whale diving behaviour, in some cases leading to lethal strandings. Diving disruption likely involves a physiological response, which remains poorly understood. Beaked whales may exceed their aerobic dive limit during long-duration deep-foraging dives and later in those dives, during ascent, initiate a unique strong gait (B-strokes), hypothesized to recruit anaerobic fast-twitch fibres. We compared the use of B-strokes during exposed and unexposed dives in four species of beaked whales. Contrasting the highly context-specific use of B-strokes during ascents from deep-dives in baseline conditions, during sonar exposure, B-strokes were used during descent and ascent phases of both deep and shallow dives. B-stroke onset occurred during all sonar exposure periods with levels above 100 dB re 1 µPa that lasted more than three minutes. The use of B-strokes during descent indicates these animals start using an oxygen-saving strategy earlier in exposed (16 ± 13 min) vs. unexposed dives (33 ± 14 min). This change in swimming gait when exposed to an external threat likely evolved to enable flexible escape responses from predators. However, if prolonged, such responses could lead to physiological changes that contribute to gas bubble formation and growth that could lead to animals stranding.

**Supplementary Information:**

The online version contains supplementary material available at 10.1038/s41598-025-22490-5.

## Introduction

Increasing anthropogenic noise in the ocean is an issue of major concern. There is growing evidence of behavioural, acoustic and physiological responses of threatened and protected cetacean species to anthropogenic sounds^1–3^. Whales may show a behavioural response that is consistent with a predator avoidance response by increasing swimming speed and dive duration, avoiding high noise areas, and ceasing foraging^4–13^. These responses often result in elevated energy expenditure and decreased energy gain, with potential implications for individual fitness and population dynamics^14,15^.

Beaked whales (Family: *Ziphiidae*) are a particularly sensitive cetacean species group^2^ as evidenced by several atypical mass strandings occurring in close temporal and spatial proximity to naval sonar exercises^16^. Despite extensive research efforts, the precise mechanism underlying these strandings remains poorly understood. However, it is likely that disruptive behavioural responses, coupled with subsequent physiological alterations play a pivotal role^17–21^. Interestingly, in narwhals (*Monodon monoceros*) exposed to airgun sounds, heart rate was lower even though stroking effort was greater^22^, indicating that natural energy-saving dive responses of cetaceans may be accentuated in the presence of a stressor. While narwhals could be restrained for instrumentation, this procedure is not feasible with beaked whales, and current technology does not permit heart rate measurements in beaked whales.

Beaked whales perform deep, long-duration foraging dives (typically greater than 450 m and 30 min), and shorter and shallower dives (< 450 m for < 22 min)^23–26^. While Blainville’s beaked whales (*Mesoplodon densirostris*), goose-beaked whales (*Ziphius cavirostris*) and Baird’s beaked whales (*Berardius bairdii*) do not seem to forage during these shallow dives, northern bottlenose whales (*Hyperoodon ampullatus*) do^6,24,27,28^. Although the precise function of these shallow dives remains elusive, they have been hypothesized to serve various purposes, including digestion, lactic acid processing, gas exchange and predator avoidance^29–31^. Tyack et al.^24^ estimated the calculated aerobic dive limit (cADL) through physiological models to be 33 min and 25 min for Cuvier’s and Blainville’s beaked whales, respectively. Joyce et al.^32^ similarly hypothesized that beaked whales seem to exceed their calculated aerobic dive limit regularly during deep dives. However, recent studies suggest that the ADL for Cuvier’s beaked whale should be closer to 77 min^33^, and that beaked whales possess morphological and biochemical specializations that likely extend their ADL^34–36^. They are expected to have a dive response that includes bradycardia and peripheral vasoconstriction to conserve available O_2_ during dives similar to better studied marine mammals^37^, though the extent of this response in beaked whales is not known.

In addition, Martín López et al.^38^ observed that during the ascent phases of deep dives, beaked whales produce high-power strokes (‘type-B’ strokes) as part of a unique mixed swimming gait. The additional acceleration generated by B-strokes did not lead to faster ascents, but rather enabled brief glides, which may improve the overall efficiency of this gait. These faster, stronger, and therefore more energetically demanding fluke strokes have only been described in beaked whale species^38,39^. Their occurrence towards the end of long dives suggests that B-strokes may recruit anaerobically-powered fast-twitch fibres that comprise ∼80% of swimming muscles in Blainville’s beaked whales^34,38^; something unique to the beaked whale family, thus prolonging foraging time at depth. The use of these fibres may help conserve available O_2_ for the heart and brain but result in in lactate formation and decreased pH. Even though anaerobic metabolism does not produce CO₂ metabolically, the acidosis it causes leads to more CO₂ being released from bicarbonate buffering. This may become a liability, since elevated CO_2_ may stimulate bubble formation, as demonstrated in the experiments performed on dead animals by^40^. The growth of gas nuclei depends on the rate of diffusion. Thus, elevated CO_2_, with high CO_2_ diffusion rate, increases gas bubble growth^40,41^. These early studies indicate that elevated levels of CO_2_ may facilitate diffusion and enhance bubble growth.

Beaked whales show consistent behavioural responses when exposed to simulated or real mid frequency active (MFA) sonar at relatively low received levels (100–130 dB re. 1 µPa). The documented responses consist of a range of avoidance behaviours including a halt in click production associated with foraging and increased swimming speed, dive duration, and dive depth while moving away from the sound source^4,6,10,27,42,43^. This shows that beaked whales respond to a perceived threat from sonar by diving deeper, longer and with greater locomotory effort, which may cause individuals to transition into an abnormal physiological state^20,21^. Such alterations could disrupt the management of gas stores accumulated during long dives^17,18,31^, possibly to the extent of causing individuals to strand^44^. These behavioural changes, combined with pathological factors such as the presence of fat/gas emboli in stranded beaked whales, have prompted suggestions that these marine mammals could experience decompression sickness if they deviate significantly from their typical gas management routine, perhaps due to stress or a pronounced behavioural response to noise exposure^17,31,44^.

The physiological process underlying this route to pathology, however, remains poorly understood. Beaked whales demonstrate exceptional capacity to buffer oxygen-deprivation during long-duration breath-hold dives, and it remains unclear which chain of events can lead to whale’s stranding during behavioural response. Here, we investigate the hypothesis that beaked whales alter their use of B-strokes during sonar exposure, thereby altering their natural patterns of oxygen use, CO_2_ storage and metabolic activity. We compare diving behaviour during MFA sonar exposed and unexposed dives in four different beaked whale species: Blainville’s beaked whales*,* goose-beaked whales, Baird’s beaked whales and northern bottlenose whales.

## Material and methods

### Subjects

The beaked whale data for this research were collected as part of several Behavioural Response Studies (BRS), investigating the response to experimental naval sonar exposure in beaked whales^4,6,27,42,43,45^ (Table 1). Specifically, we analysed the available BRS beaked whale data (n) and up to 10 baseline data records (N) per species: Blainville’s beaked whales (Md n = 2, N = 10), goose-beaked whales (Zc n = 3, N = 10), Baird’s beaked whales (Bb n = 1, N = 0) and northern bottlenose whales (Ha n = 4, N = 10),

**Table 1:** Field research experiment summary for baseline and behavioural response study data.

Species	Field research experiment					
	Baseline data			BRS data		
	N	Location and year	References	n	Project, location and year	References
Blainville’s beaked whale	10	El Hierro, Canary Islands (Spain; 2003–2008) Abaco and Andros Islands (The Bahamas, USA; 2007–2017)	^24,47^	2	AUTEC BRS-project. Waters of Andros Island (The Bahamas, USA; 2007–2008)	^4^
Goose-beaked whale	10	Ligurian Sea, (Italy; 2004–2006) Azores (Portugal; 2015–2018) Southern California Bight (USA;2013)	^24,48,49^	3	SOCAL BRS- project Southern California Bight (USA; 2010–2013)	^42,45^
Northern bottlenose whale	10	Jan Mayen (Norway; 2013–2016)	^6,8–10,43,50^	4	3S-project Jan Mayen (Norway; 2013–2016)	^6,43^
Baird’s beaked whale	0			1	SOCAL-BRS- project California, (USA; 2012)	^27^

N, number of baseline deployments; n, number of BRS deployments.

### Data collection

All data were collected with suction cup attached multi-sensor tags (DTAGs)^46^; which included two hydrophones sampled at 96, 192 or 240 kHz depending on the DTAG version, and a pressure sensor, triaxial accelerometers and triaxial magnetometers that were all sampled at 50 or 200 Hz with 5 Hz and 50 Hz anti-alias filters, respectively. These sensor streams were decimated to a common 25 Hz sampling rate in post processing. Once the tag was attached, the animal was either left unexposed (baseline deployment) or a protocol for a Controlled Exposure Experiment (CEE) was followed. This CEE protocol was similar for all projects, with slight differences (Table S1). A baseline pre-exposure period of 1–7 h was followed by the exposure period in which the ship carrying the sonar source gradually approached the position of the tagged whale or gradually increased the transmitted source level to achieve an escalation of the received Sound Pressure Levels (SPL) from initial values of 66–112 dB to maximum levels of 99–151 dB re 1 µPa (Root Mean Square, RMS values) (Table S1). Complete details of the experimental procedure and calculations of the received RMS SPL, cumulative Sound Exposure Level (SELcum), as well as the whale response to the exposure can be found in the references within Table 1.

### Data processing

The triaxial accelerometer and magnetometer signals were rotated in post-processing to correct for the orientation of the tag on the whale, which was estimated at each surfacing from the stereotypical movements during respiration^46^. Beaked whales perform long, deep foraging dives interspersed with extended periods of shorter shallow dives^51^, which in the case of Md and Zc do not appear to include foraging^24^. By pooling all dives deeper than 20 m of all species together; we define deep dives as all of those with maximum depth greater than 400 m and shallow dives as all those dives with a maximum depth greater than 20 m and less than 400 m.

To identify individual locomotory strokes, the dominant stroke frequency (sensu Sato et al.^52^) was first estimated for each animal as the peak frequency in the spectral average (256-point FFT, 50% overlap) of the dorso-ventral accelerometer signal. A symmetric Finite Impulse Response low-pass filter with a cut-off frequency of 0.4 of the dominant stroke frequency was used to separate stroking from the low frequency orientation postural signals in both the accelerometer and magnetometer sensors as described in Martín López et al.^38^. Individual half-strokes were detected in the body rotation signal. To separate regular (A-strokes) from strong and short-duration strokes (B-strokes), histograms of the RMS heave specific acceleration signal for each half stroke were plotted for each whale^38^.

To differentiate between dive phases, we calculated the pitch angle from the low-pass-filtered acceleration data, with negative pitch angles indicating descent. Following the methodology of Miller et al.^53^, we defined descents as the interval from the start of the dive until the pitch angle first exceeded 0 degrees. Ascents were identified as beginning at the last instance in the dive where a sustained downward pitch (< 0 degrees) was observed. The reliability of this method was verified visually, and corrections were made in instances where a brief descent occurred during the ascent phase or vice versa. For each dive exceeding 20 m, we calculated: dive duration, maximum depth, time to previous deep dive (calculated as the time interval between the start of the current dive, and the end of the previous deep dive), duration of the descent and ascent phases, the number of regular and B-strokes in each descent and ascent phase (fluke stroke count) and the proportion of regular and B-strokes in each dive phase. The proportion of B-strokes in each phase was determined by dividing the total number of B-strokes in each phase by the total number of strokes (regular strokes plus B-strokes). For those dives with B-strokes present, we also calculated the onset time of B-strokes as the elapsed time from the beginning of each dive until the first dive minute in which the B-stroke rate in that minutes was greater than or equal to the median B-strokes-per-minute for all ascents of each whale (sensu Martín López et al.^38^).

Rapid manoeuvring during foraging in the bottom phase makes it difficult to detect individual fluke-strokes and so strokes were not identified in this phase^38^. md08_271a has been excluded from this analysis as the CEE occurs during the bottom phase where we do not have stroke rate information, and the tag fell off just before the ascent phase started. md07_245a was exposed to simulated MFAs and 6 h later to killer whale (*Orcinus orca)* playbacks. To be consistent with the analysis of how sonar affects the presence and onset of B-strokes, we excluded from the analysis all data after the start of the killer whale playback (11.5 h). We also classified each dive as baseline or pre-exposure, exposed, or post-exposure dives. Baseline dives were those dives from tag deployments that were not exposed to sonar. For tag deployments that were subject to sonar exposures, pre-exposure dives were those dives that occur prior to any sonar exposures. This provides baseline data at the level of the individual tag deployment, which is useful for controlling variation in a specific individual, social and environmental context. Exposed dives were those dives during which sonar pings were recorded on the tag, while post- exposure dives were those that occur after those exposed dives.

### Statistical analysis

We used two different statistical models to assess the impact of sonar exposure on the gait biomechanics of beaked whales. The first modelled the presence-absence of B-strokes at the dive level, defining presence as those dives with at least the median ascent rate of B-strokes for each whale. The second modelled B-stroke proportion at the level of sonar inter-ping intervals for each CEE deployment (n = 10). The proportion of B-strokes per ping interval was calculated by dividing the total number of B-strokes in each interval by the total number of strokes, comprising of both regular and B-strokes. Inter-ping intervals ranged from 20 to 30 s depending on the CEE deployment. To account for baseline conditions (i.e., baseline sound levels and presence of B-strokes during the ascent phase of long dives) we selected pre-exposure data by analysing the same ping interval time section (i.e., 20, 25 or 30-s sections) for each deployment just before the CEE occurred (e.g. for 30 min of sonar exposure with a ping interval of 25 s, we selected the previous 30 min to the first sonar ping, to calculate the parameters to include in the model within 25 s). During these pre-exposure intervals, we set the SPL to a reference level of 60 dB re 1 µPa, which was 6 dB below the lowest measured received SPL, to represent a below-ambient level that would be inaudible to the whales^54^. For each ping-to-ping interval, during both pre-exposure and exposure, we extracted the following parameters: *dive type* (deep vs. shallow), *dive depth*, *dive phase* (descent vs. ascent), RMS received *SPL* and the *proportion of B-strokes* produced. We chose to use a Bayesian approach to facilitate handling of missing values in the response variable. B-strokes could not be detected, and therefore led to missing values in the B-stroke proportion, during the bottom phase (the time between the end of the descent and the start of the ascent of each dive) and surface periods (periods at the surface and during immersions shallower than 20 m).

Generalized Additive Mixed Modelling (GAMMs) was selected as the primary statistical approach to allow for flexible relationships to be estimated alongside random effects. We used the *gamm4* R package to fit a Bernoulli model to the presence-absence of B-strokes. *gamm4* uses the *mgcv* package for gams and *lmer* for the mixed modelling part. For the proportion of B-strokes, however, we used a beta distribution and fitted through the Bayesian *inlabru* package.

The B-stroke presence-absence model selection was performed in two steps. First, we performed model selection to identify predictors for *B-stroke presence* in baseline behaviour. Once this model was selected, we included sonar exposure in the best baseline model through either the *presence-absence of sonar* in the dive or the *cumulative SEL at the end of the dive*. The baseline model included a random effect for *TagID* nested within *species* and considered all combinations of up to three covariates: *dive duration*, a *regular fluke stroke rate* metric (number of regular fluke strokes either during descent or during both descent and ascent) and a previous dive metric (either *previous dive duration*, or *number of B-strokes in previous dive*). The best, most parsimonious model was selected as the simplest model within 2 Akaike Information Criterion (AIC) units of the lowest-AIC model. A second set of models considered their inclusion as smooth covariates instead. Here, alternative full models were fitted and backwards selection was carried out using shrinkage smooths. These penalize the smooth to zero when it is not supported.

The ping-to-ping B-strokes proportion model included a *TagID* random effect and first order autocorrelation structure for each deployment. We included a second-order random walk latent field to capture the expected non-linear effect of *SPL* in the *proportion of B-strokes*. Additionally, we tested the impact of *dive type* and *dive phase* as these are known to affect the presence of B-strokes and the interaction between these two variables given that B-strokes are more likely to occur during the ascent of deep dives in baseline data^38^. Model selection was based on the Watanabe Akaike Information Criterion (WAIC)^55^. Unlike AIC, which is based on a point estimate of the model parameters, WAIC averages over the posterior distribution, providing a more fully Bayesian approach to model selection. The lower the WAIC, the better the model’s predictive accuracy.

### Ethics and permissions

All experiments were performed in accordance with relevant guidelines and regulations for studying wild animals such as the ARRIVE guidelines (https://arriveguidelines.org). Data were gathered with ethics authorization of the U.S. National Marine Fisheries Service (permits #1121-1900, #14534, #981-1578, #981-1578-02 and #981-1707-00), the Government of The Bahamas (permits #01/09, #12A, #02/07 and #02/08), the Norwegian Animal Research Authority (permit #S2011/38782 and #2015/23222), the Icelandic Ministry of Fisheries, the Channel Islands National Marine Sanctuary (permits #2010-003 and #2010-004). The experimental research was approved by the Woods Hole Oceanographic Institution, the BMMRO’s Institutional Animal Care and Use Committee, the US Animal Welfare Act, the Animal Welfare and Ethics Committee of the University of St Andrews (U.K.), the Governments of Spain and the Canary Islands, and by the Secretaria Regional do Mar, Ciência e Technologia, Direção Regional dos Assuntos do Mar (Azores, Portugal).

## Results

**Fig. 1:**
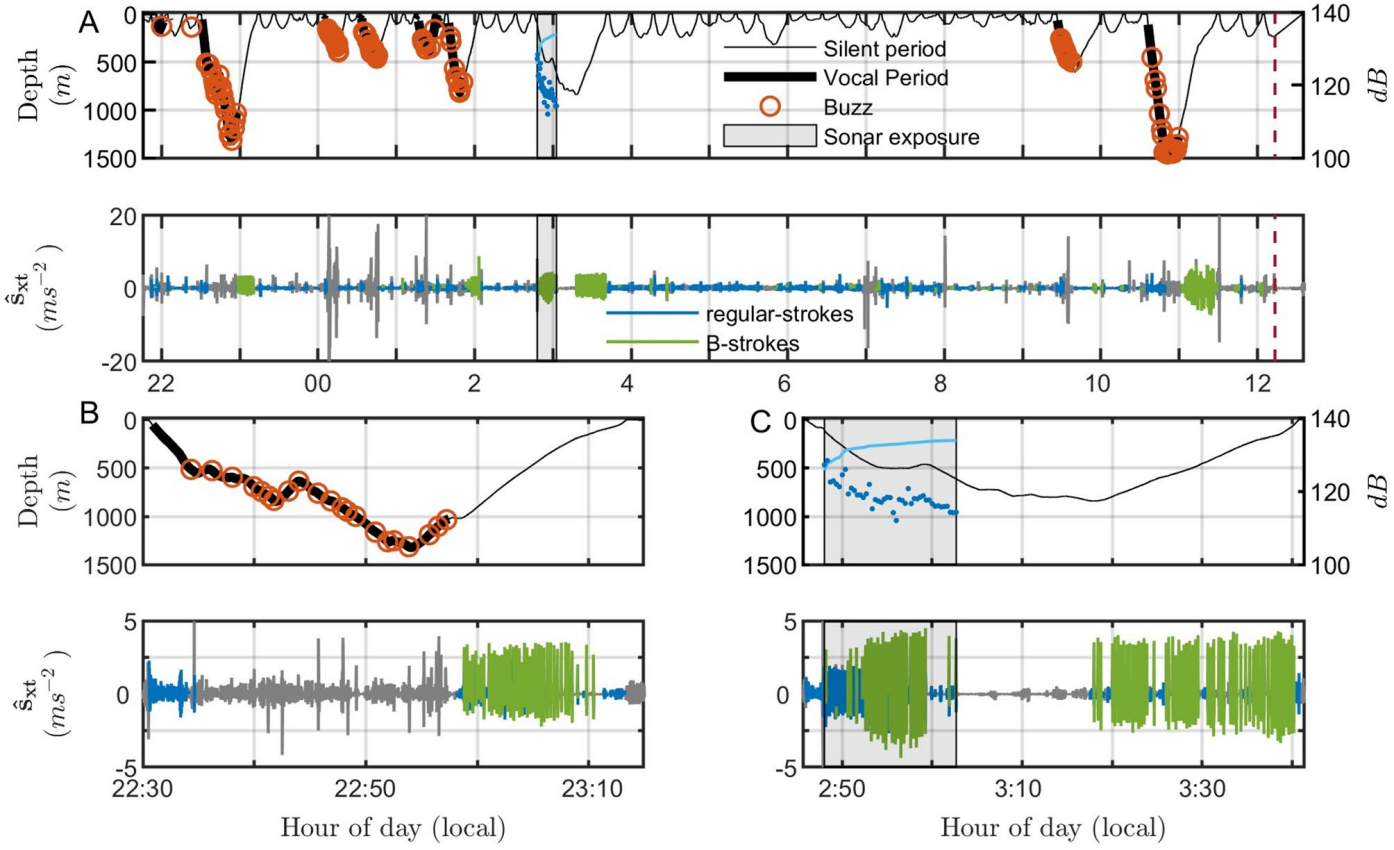
Beaked whale dive and stroking behaviour in baseline and exposure conditions. (**A**) Upper panel: Time-depth record of tagged bottlenose whale ha15_179b with the timing of the sonar exposure period lightly shaded between vertical lines and received levels of the sonar with ping-by-ping SPL (in dB re 1 µPa) shown as dark blue dots and cumulative sound exposure level (in dB re 1 µPa^2^ s) shown as a solid light blue line. Sounds are marked by colour: bolded black line shows periods when the tagged whale was producing foraging echolocation clicks and red circles indicate buzzes (i.e., likely foraging attempts); lower panel: surge specific acceleration $({\widehat{s}}_{xt})$ in ms^−2^ with regular and B-strokes coloured in blue and green, respectively during the descent and ascent phase. Bottom phase is coloured in grey. (**B**) Zoomed view of the dive profile (upper panel) and ${\widehat{s}}_{xt}$ (lower panel) during the first baseline deep dive (1320 m). (**C**) Zoomed view of the dive profile (upper panel) and ${\widehat{s}}_{xt}$ (lower panel) during the controlled exposure experiment deep dive (840 m).

Beaked whale diving patterns in baseline conditions typically consist of deep foraging dives interspersed with a series of shallow dives (Fig. 1). In total we analysed 235 deep dives (DD) and 776 shallow dives (SD) with a mean (± sd) duration of 40.5 ± 17.8 (DD) and 11.9 ± 6.4 (SD) min, respectively. However, this duration varied with species (Table 2).

**Table 2:** Deep (DD) and shallow (SD) dive statistics during pre-exposure and baseline dives, sonar exposed dives and post-exposure dives of all species.

Species ID	Dive type	Parameter	Condition		
			Pre-exposure	Exposure	Post-exposure
Md	DD	Dives analysed	57	1	0
		Dive duration (min)	50 ± 10	52	–
		Ascent duration (min)	18 ± 5	25	–
		Descent duration (min)	8 ± 2	12	–
		Dives with B-stroke onset	54	2	–
		B-stroke onset (min)	35 ± 8	42	–
	SD	Dives analysed	326	0	8
		Dive duration (min)	11 ± 3	–	13 ± 4
		Ascent duration (min)	4 ± 3	–	3 ± 2
		Descent duration (min)	4 ± 2	–	3 ± 2
		Dives with B-stroke onset	5	–	0
		B-stroke onset (min)	12 ± 4	–	–
Zc	DD	Dives analysed	63	2	5
		Dive duration (min)	57 ± 17	78 ± 14	53 ± 22
		Ascent duration (min)	19 ± 7	43 ± 31	27 ± 8
		Descent duration (min)	11 ± 4	6 ± 5	12 ± 3
		Dives with B-stroke onset	55	2	3
		B-stroke onset (min)	46 ± 12	21 ± 6	62 ± 3
	SD	Dives analysed	171	2	27
		Dive duration (min)	17 ± 7	22 ± 2	19 ± 6
		Ascent duration (min)	7 ± 4	14 ± 6	10 ± 5
		Descent duration (min)	7 ± 4	0.5	6 ± 3
		Dives with B-stroke onset	20	2	1
		B-stroke onset (min)	16 ± 5	10 ± 11	17
Ha	DD	Dives analysed	115	3	3
		Dive duration (min)	27 ± 10	54 ± 42	44 ± 9
		Ascent duration (min)	9 ± 6	35 ± 28	21 ± 5
		Descent duration (min)	5 ± 3	2 ± 2	8 ± 5
		Dives with B-stroke onset	38	2	1
		B-stroke onset (min)	24 ± 9	7 ± 2	29
	SD	Dives analysed	272	5	80
		Dive duration (min)	10 ± 7	15 ± 9	15 ± 7
		Ascent duration (min)	3 ± 3	4 ± 2	4 ± 4
		Descent duration (min)	3 ± 3	4 ± 4	4 ± 3
		Dives with B-stroke onset	4	2	10
		B-stroke onset (min)	26 ± 8	20 ± 2	14 ± 5
Bb	DD	Dives analysed	0	1	1
		Dive duration (min)	–	51	56
		Ascent duration (min)	–	21	19
		Descent duration (min)	–	19	8
		Dives with B-stroke onset	–	1	1
		B-stroke onset (min)	–	1	50
	SD	Dives analysed	7	2	9
		Dive duration (min)	18 ± 6	18 ± 10	18 ± 8
		Ascent duration (min)	9 ± 5	8 ± 3	5 ± 4
		Descent duration (min)	6 ± 5	7 ± 9	10 ± 4
		Dives with B-stroke onset	1	2	0
		B-stroke onset (min)	14	11 ± 13	–

Values are means ± s.d for each species. Species ID represents the species scientific name initials: *Mesoplodon densirostris* (*Md*), *Ziphius cavirostris* (*Zc*), *Hyperoodon ampullatus* (*Ha*) and *Berardius bairdii* (*Bb*). Durations are given in minutes (min)

### Diving and B-strokes in baseline conditions

Baseline data for Md and Zc were quite similar in terms of dive duration and B-onset time as previously described by Martín López et al.^38^, with B-strokes produced during the ascent phase of most deep dives (147 out of 235), and in a small number of shallow dives (30 out of 776) that occurred just after a deep dive (Table 2). No B-strokes were produced during the descent phase of any deep or shallow dive. For Md, Zc and Ha, deep dive duration means were 50, 57 and 27 min, respectively. B-strokes began during the ascent phase of deep dives at mean times of 35 min (Md), 46 (Zc) and 24 min (Ha). No baseline deep dives were recorded for Bb.

### Diving and B-strokes under sonar exposure conditions

**Fig. 2:**
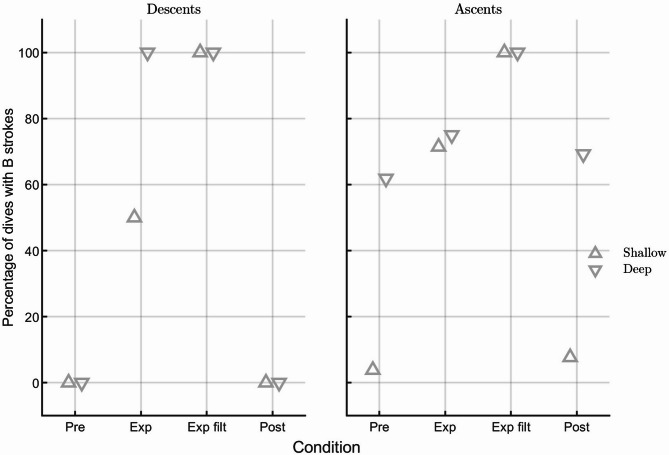
Beaked whales alter B-stroke behaviour during navy sonar exposure. Percentage of dives with B-stroke onset during dive-descent and -ascent phases for deep and shallow dives, under the three experiment phases (Pre = pre-exposure and baseline, Exp = during exposure and Post = post-exposure). Exp filt: a subset of dive phases that have been exposed with RL > 100 dB re 1 µPa for more than 3 min.

**Fig. 3:**
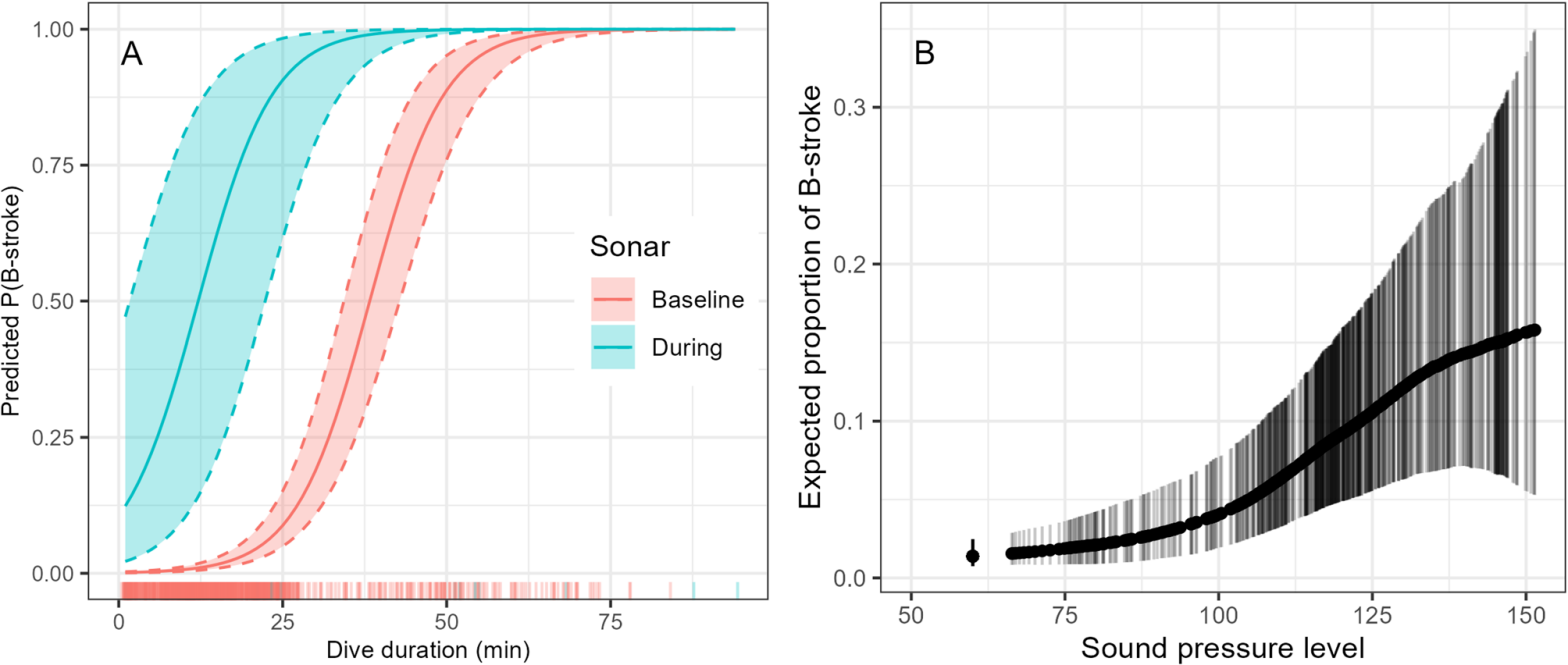
B-strokes are more likely to occur in the ascent phase during sonar exposure, especially in shorter dives, and to increase in frequency as sonar sound pressure level rises. (**A**) Predicted probability of B-stroke presence during ascent as a function of dive duration during pre-exposure baseline (pink) and sonar exposure (blue), based on Model 27 (Table S3). Shaded areas show 95% normal prediction intervals. X-axis rug plot shows data coverage, including both pre-exposure baseline and sonar exposure data. Predictions are provided given a median value for fluke stroke rate during descent and ascent. (**B**) Expected proportion of B-strokes per-ping interval at different sonar sound pressure levels (SPL) based on the selected model (M1 model from Table S4). Points refer to the mean estimate and bars represent the 95 credible intervals. Plotted estimates refer to the actual data, thus darker areas with denser lines represent received SPLs with more data. The point at 60 dB represents the expected proportion of B-strokes during pre-exposure baseline.

During exposed dives (dives during which sonar pings were recorded on the tagged whale, n = 16) beaked whales strongly altered their B-stroke behaviour, making earlier use of B-strokes than during baseline dives (mean ± sd: 15 ± 12 vs. 33 ± 14 min, respectively). Specifically beaked whales made an earlier use of B-strokes when exposed than during baseline in deep dives (mean ± sd: 16 ± 15 vs. 36 ± 13 min, respectively) as well as in shallow dives (mean ± sd: 14 ± 9 vs. 17 ± 7 min, respectively). B-strokes were employed during most deep (6 out of 7) and shallow dives (6 out of 9), and during both dive phases (descent and ascent; Figs. 1 and 2—see exposed condition). Moreover, during sonar exposure, (i) the probability of B-stroke presence during the ascent phase of short dives increased (Fig. 3A) and (ii) the proportion of B-strokes (number of B-strokes divided by the total number of strokes per ping interval) increased with received SPL (Fig. 3B).

**Fig. 4:**
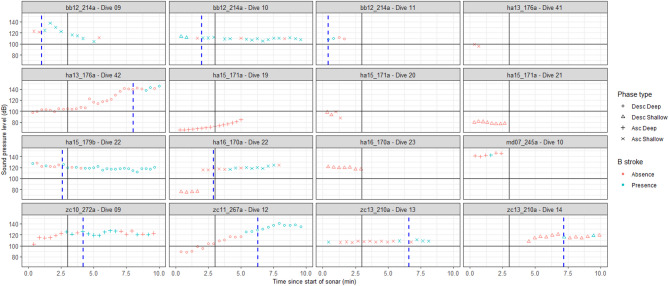
During exposed dives with received sound pressure levels above 100 dB re 1 µPa for more than three min (n = 10), all whales showed a clear response to sonar sounds, i.e., an earlier B-onset time. The plot shows the received sound pressure levels (SPL, in dB re 1 µPa) of each sonar ping for each of the 16 dives performed by nine individual whales from four beaked whale species exposed to controlled sonar. Each subplot corresponds to a specific whale and dive, with the tag ID and dive number indicated in the title. The tag ID format includes species initials, year (two digits), Julian day, and tag deployment letter. Sonar pings are represented by different symbols indicating the dive type and dive phase in which the sonar ping was heard: downward triangles (shallow dive descent), upward triangles (shallow dive ascent), circles (deep dive descent), and crosses (deep dive ascent). Symbols are coloured red if no B-strokes were detected during that ping interval, or blue if at least one B-stroke occurred. The B-onset time is indicated by a dashed blue line. Note that some dives show B-strokes without a defined B-onset, as B-onset time is estimated as the elapsed time from the beginning of each dive until the first minute with at least the median ascent rate of B-strokes for each whale. SPL values are plotted for up to 11 min of each dive for ease of visualization. Each subplot includes markers for 100 dB re 1 µPa SPL and three min (i.e., a black horizontal line and a black vertical line, respectively) since the start of sonar for easy reference. For a detailed description of B-stroke occurrence in response to sonar, see Table S2.

During exposed dives with received SPLs above 100 dB re 1 µPa for more than three min (n = 10), all whales showed a clear response to sonar sounds (Fig. 4, Table S2) (see exposed filtered condition in Fig. 2), with B-strokes produced in all phases and dive types. During these specific exposed dives, the onset of B-strokes occurred earlier in the dive than during baseline dives (mean ± sd: 14 ± 10 vs. 33 ± 14 min, respectively; Fig. 1). Specifically, the onset time of B-strokes for exposed deep dives vs. baseline deep dives was 14 ± 8 vs. 36 ± 13 min, respectively. Specific times for each species are given in Table 2.

In addition to received SPL, the duration over which the animal was exposed to the sonar sound affected the B-stroke onset. Animals exposed to a received level below 100 dB re 1 µPa (ha15_171a) and/or less than two min (md07_245a and ha16_170a), did not show a clear B-stroke response to the exposure. E.g., during the last exposed shallow dive ascent of ha16_170a, no B-strokes were present (exposure time > 100 dB was < 2 min). However, when exposed to SPL above 100 dB re 1 µPa for longer time during the ascent phase of the previous shallow dive, the individual did perform B-strokes (see details in Supplementary Table S2). All whales exposed to SPL above 100 dB re 1 µPa for longer than three min performed B-strokes within that dive phase. The B-stroke onset within the dive phase occurred from one to eight min after the start of the sonar sound in that dive phase.

It is noteworthy that while no behavioural response to sonar was observed for zc13_210a during the initially published research^45^, this animal did use B-strokes during the ascent phase of a shallow dive and during both phases of the following shallow dive during which sonar pings were recorded.

### Effect of sonar on B-stroke occurrence

Statistical modelling supported increased B-stroke occurrence during sonar exposures compared to baseline. In the B-stroke presence-absence analysis, the most parsimonious baseline model included *dive duration*, *regular fluke stroke rate* during both descent and ascent, and *previous dive duration*. All three variables were also retained by the shrinkage smooths but estimated near-linear relationships for all three variables,

When fitted to both baseline and exposure data, the inclusion of an additional sonar covariate was supported both by the AIC model selection (Table S3) and hypothesis-testing at 5% significance level (Wald-test for sonar presence/absence covariate: df = 1, χ^2^ = 10.1, *p* = 0.002, Wald-like test for cumulative SEL smooth covariate: df = 1.1, χ^2^ = 571.8, *p* < 0.001). Despite support for the *previous dive duration* covariate in baseline model selection (Table S3), the covariate gained only weak support in exposure models 23–25 (Table S3) fitted to both pre-exposure and exposure data (χ^2^ = 3.3–4.0, *p* = 0.04–0.06). Inference on sonar presence/absence effects was therefore based on a simpler exposure model excluding this covariate. The probability of B-stroke presence during the ascent phase of short dives significantly increased during sonar exposure dives (Fig. 3A).

The increase in B-strokes during sonar exposure was associated with the SPL received from the sonar source (Fig. 3B, Table S4), in deep as well as shallow dives, and in both the descent and ascent phases (Table S4, model M0) (Supplementary Fig. S1). The higher the received sonar SPL, the greater the expected proportion of B-strokes, reaching an asymptote at around 135 dB re 1 µPa (Fig. 3B).

## Discussion

Within one to eight minutes following sonar exposure, beaked whales responded consistently by initiating a strong and short-duration gait likely to be powered by anaerobic fast-twitch fibres, irrespective of diving phase (descent or ascent) or whether a dive was deep or shallow. This response occurred consistently across all four beaked whale species tested when exposed to sound pressure levels above 100 dB re 1 µPa for more than three min (7 out of 9 whales, Fig. 4). This is in clear contrast with the baseline use of B-strokes, in which they are largely restricted to the point in ascents from long-duration deep foraging dives at which their oxygen stores are expected to dwindle. Occasional B-strokes occurred during the ascent phase of some shallow dives (30 out of 776 dives) in baseline conditions, all of which immediately followed a deep dive, as reported previously in Martín López et al.^38^. These results indicate that when exposed to sonar, beaked whales switch to a powerful gait that likely requires less oxygen, at the cost of increased lactic acid build-up, particularly if this alternative gait is paired with a flight response with faster speeds as shown in prior studies^42,43^. These B-strokes occur not only earlier in the dive, but also in behavioural contexts in which this gait was never observed in a large number of baseline records (N = 1011 dives).

Received SPL of the sonar, as well as the duration that the animal was exposed to the sonar sound, affected B-stroke onset. Moreover, the presence of B-strokes during ascent increased in sonar-exposed dives beyond what was expected by dive duration, and the proportion of fluke strokes employing the B-stroke gait during a dive following sonar exposure also correlated positively with received SPL. Hence, louder and longer sonar exposure increased the likelihood of this alternative locomotory strategy being employed. Taken together, these results provide strong support for our hypothesis that navy sonar exposure induces a distinct locomotor response with potential physiological consequences to beaked whales.

The question of how anthropogenic activities disturb animals is central in conservation studies. Beaked whales died during the mass strandings in temporal and spatial association with Navy sonar in the early 2000s in The Bahamas and Canary Islands. These animals presented gas bubble lesions and fat emboli suggesting a series of pathophysiological events that occur in vivo^17,56^. Since these mass strandings, baseline as well as BRS data have been collected in order to elucidate the baseline behaviour of beaked whales and how these animals change this behaviour in response to sonar sounds. In the current study, we used all available and suitable beaked whale behavioural response data available to date. Whereas this remains a comparably small exposure dataset (7 deep and 9 shallow dives exposed to sonar exposed vs. 235 deep and 776 shallow baseline dives from 9 tag deployments), the consistent and clear locomotory response to sonar across the different beaked whale species clearly identified this effect of sonar exposure.

Beaked whales have been shown to respond to sonar by strong avoidance, which typically involves longer and deeper dives while moving away from the source, increased swim speed, and cessation of foraging^4,6,27,42^. Some of these responses, such as the cessation of foraging, can persist for up to seven h following the end of sonar exposure^6,42^. The switch to a strong gait reported here matches and further augments the signature of a strong avoidance response. Exposed whales increasingly employed B-strokes with increasing received SPL (Fig. 3B). The altered gait did not persist in post-exposure dives; the percentage of dives with B-stroke onset times during descent and ascent phases for both dive types (shallow and deep) that were performed during post-exposure experiments were similar to those performed during pre-exposure dives (see Fig. 2). We propose that as the whales cannot predict how long they need to continue to avoid a potential threat and persist in an energy-consuming flight response, when exposed to SPL above 100 dB re 1 µPa for more than three min, they aim to conserve their available oxygen for vital organs by switching to a combination of aerobic and anaerobic locomotor modes, i.e., normal strokes interspersed with short-duration and strong B-strokes.

During baseline diving in beaked whales, deep and long dives are typically but not always interspersed with several repeated shallow and short dives^24,30,33^. These shallow dives are thought to serve various purposes, such as digestion, gas exchange, predator avoidance and to recover from previous deep dives, in which accumulated CO_2_ and lactic acid is processed^24,29–31^. The timing of a sonar exposure may disrupt this dive pattern. Long periods of B-strokes, as a consequence of an earlier B-stroke onset, with a combination of aerobic and anaerobic metabolism, result in accumulation of lactic acid during the dive, which interacts with the bicarbonate system to release more CO_2_^40^. Thus, depending on the timing of when beaked whales are exposed to sonar within a dive series, an altered gait may result in excessive levels of both CO_2_ and lactate. For example, if an animal was planning to perform a shallow dive, during which lactic acid would be processed and is exposed to sonar, the changes in swimming gait would result in an increase in the lactic acid concentration, and CO_2_ in their muscles^57^. If this behavioural change occurs in the first shallow dive after a deep dive, where lactate and CO_2_ build up are likely significant, the effect may be enhanced. Past work, on dead animals in which the active transport of lactate is no longer functional, has shown that excessive blood and tissue CO_2_ increases the risk of symptomatic gas bubble formation and growth^40^, that could result in the animals stranding. The altered use of B-strokes may thus be a physiological pathway specific to beaked whales which increases stranding-risk from navy sonar.

Frequent and continued exposure to navy sonar may exacerbate physiological effects on individuals over time, although it could alternatively be that after decades of being exposed, animals habituate to sonar or move away from their preferred habitat. Resident beaked whales at southern California do not appear to habituate to sonar; instead, they continue to show strong, potentially costly behavioral responses, with foraging disruption during and after sonar use^10^. On the other hand, resident beaked whales at AUTEC are considered to have habituated to repeated sonar exposures, exhibiting consistent avoidance behavior whereby individuals temporarily leave their preferred habitat during sonar operations and then return to these habitats after intense sonar operations^4^. This temporary displacement, observed on naval ranges such as those near Andros Island, The Bahamas, can be energetically costly, potentially impacting foraging and overall fitness^4,12,32,58,59^. Thus, despite repeated exposure to navy sonar, beaked whales in these areas continue to show strong behavioral responses to sonar.

A process enhancing the potential physiological effects of unplanned use of B-strokes is heart-rate regulation during breath hold dives. Measurements of heart rate in cetaceans are challenging to obtain, but show striking results for the few species for which this has been successful. For example, narwhals exposed to seismic airgun pulses and vessel noise showed a prolongation of high intensity activity that coincided with an intense bradycardia, i.e., extremely low heart rates^22^. The effect of changes in heart rate is difficult to predict and depends on blood flow distribution. A reduction in heart rate, and perfusion of peripheral tissues, is the dive-response mechanism^37^ to conserve available O_2_ for vital organs such as the heart and brain, making muscle tissue more hypoxic. Initially this does not create a problem, as the muscle relies on endogenous O_2_ (O_2_ bound to myoglobin) for aerobic metabolism. Longer duration or higher activity, however, will increase utilization rate and the muscle will eventually run out of O_2_. The aerobic metabolism and reduced blood flow will result in an elevated concentration of CO_2_ in the muscle^19,40,60^. In addition, reduced heart rate during recovery dives has also been associated with incomplete recovery of muscle and blood O_2_ stores^61^ which may further limit the aerobic dive duration during subsequent dives. Interestingly, long-finned pilot whales, another deep-diving species that show avoidance of navy sonar^62^, but with different muscle fibre profile to beaked whales supporting high-activity events^34^, display reduced respiration efforts during sonar exposures compared to rates of recovery expected based on diving history^63^. Taken together, these literature results show that the escape responses of deep-diving cetaceans are associated with physiological trade-offs, with consequences that may differ by the species’ adaptations to manage diving metabolism and recovery.

In conclusion, we report a sonar-induced locomotory response in beaked whales that is likely to have physiological implications for the management of gas stores during breath-hold diving. The switch in swimming gait suggests a need to conserve oxygen while moving away from the source, but may have more severe physiological consequences when prolonged, and for individuals that are already near the physiological limits of their diving performance. In such contexts, this locomotory response could be another risk factor that has contributed to the mass stranding of beaked whales as a result of naval sonar exposure.

## Supplementary Information

Supplementary Information.

## Data Availability

Data for this paper will be deposited in the Dryad Digital Repository 10.5061/dryad.dv41ns28t. The data sharing policy in general concerns the minimal dataset that supports the central findings of a published study. Lucía Martina Martín López should be contacted if someone wants to request the data from this study at luciamartinaml@gmail.com.

## Abbreviations

AIC: Akaike information criterion
Bb: *Berardius bairdii*, Baird’s beaked whales
BRS: Behavioural response study
CEE: Controlled exposure experiment
DD: Deep dive: a dive greater than 400 m deep
Ha: *Hyperoodon ampullatus*, northern bottlenose whales
Md: *Mesoplodon densirostris*, Blainville’s beaked whales
n: Number of behavioural response or exposed deployments
N: Number of baseline or unexposed deployments
RMS: Root mean square
sd: Standard deviation
SD: Shallow dive, a dive greater than 20 m and less than 400 m deep
SELcum: Cumulative sound exposure level, measured in dB re 1 µPa
SPL: Sound pressure level measured in dB re 1 µPa
${\widehat{s}}_{xt}$: Surge specific acceleration measured in ms^−2^
WAIC: Watanabe Akaike information criterion
Zc: *Ziphius cavirostris*, goose-beaked whales
