# Supplementary material for: Naval sonar induces an anaerobic swimming gait in beaked whales
Source: Sci Rep. 2025 Nov 5;15:38686. doi: 10.1038/s41598-025-22490-5 (PMC12589398; doi:10.1038/s41598-025-22490-5)
Supplement: Supplementary file 1 — Supplementary Information. [file 41598_2025_22490_MOESM1_ESM.pdf]

Supplementary Information

Title: Naval sonar induces an anaerobic swimming gait in beaked whales

L.M. Martín López[1,2,3], S. Isojunno[1], D. Cade[4], K. Colson[3], I. Paradinas[2,5], P.J.O. Miller[1], A. Fahlman[6,7,8], L.S. Hickmott[1,9] and F. Visser[3,10].

[1]SMRU, Scottish Oceans Institute, University of St Andrews, St Andrews, Fife KY16 8LB, UK. [2]Asociación IPAR Perspective, 33508, El Allende Llanes, Spain. [3]Hopkins Marine Station, Stanford University, 93950 California, USA. [4]Kelp Marine Research, 1624 CJ Hoorn, the Netherlands. [5]AZTI, Txatxarramendi Ugartea z/g, Sukarrieta, Bizkaia 48395, Spain. [6]Fundación Oceanogràfic, Valencia, Spain. [7]Global Diving Research SL, Sanlucar de Barrameda, Spain. [8]IFM, Linkoping University, Linkoping, Sweden. [9]Open Ocean Consulting, Hampshire GU32 2EY, United Kingdom. [10]Royal Netherlands Institute for Sea Research, PO Box 59, 1790 AB, Den Burg, the Netherlands.

Correspondence author: Lucía Martina Martín López- email: luciamartinaml@gmail.com

Table S1: Description of experimental conditions used in behavioural response field studies included in this research: TagID: the code comprises the scientific species name initials, the year (two digits), the Julian day, and the tag deployment of the day (a single letter); sound source type: simulated mid-frequency active (MFA) sonar (1-2kHz, 2-5 kHz, 3–4 kHz, 3.5-4 kHz), killer whale playback sound (2-5 kHz), pseudo-random-noise (PRN, 2-5 kHz), real mid-frequency active (MFA) sonar (3–4 kHz); initial and maximum received Root Mean Square (RMS) Sound Pressure Level (SPL) in dB re 1 µPa; number of sonar pings or pulses (ping n); sound exposure duration (sound dur) in minutes; total deployment duration (tag dur) in hours; baseline duration prior to sound exposure (baseline dur) in hours; and description of behavioural responses to sonar as reported by Tyack et al. [1], DeRuiter et al. [2], DoN [3], Miller et al. [4], Wensveen et al. [5] and Stimpert et al. [6]. The code for the responses goes as follows: 1: cease vocalizing, 2: move away from sound source, 3: long shallow ascent, 4: increase in speed and body acceleration, 5: decrease in speed and body acceleration, 6: move towards the sound source,

TagID	Sound	RMS SPL (dB re 1 µPa)		Ping (n)	Sound dur (min)	Tag dur (hour)	Baseline dur (hour)	Behavioural response to sonar
		Start	max					
md07_245a	Simulated MFA	80	146	31	~ 13	17.5	3.5	1,2,3 [1]
md07_245a	Killer whale							1,2,3 [1]
md08_271a	PRN	86	142	20	~ 8	3.8	3.6	1,3 Tag fell off during the shallow ascent of the sonar exposed deep dive [1].
zc10_272a	Simulated MFA	87	138	72	~ 30	8.8	4.9	1,4 [2]
zc11_267a	Simulated MFA	91	144	72	~ 30	21.2	9.2	1,5[2] [2]
zc13_210a	Real MFA	107	124	120	~ 60	8.1	7.2	No response was observed [3] Tag fell off just at the end of the last sonar

								ping, during the bottom phase of a deep dive.
ha13_176a	Simulated MFA	96	151	86	~ 32	18.3	10.7	1,2. Unusual deep dive during the exposure, the deepest recorded in this species [4].
ha15_171a	Low-level LFA (1-2 kHz)	66	99	46	~ 15	9.4	3.7	1,6 [5]
ha15_179b	LFA	112	128	45	~ 15	14.4	5	1,2,4 [5].
ha16_170a	Simulated MFA	75	128	71	~ 30	13.3	5.2	1,2 [5].
bb12_214a	Simulated MFA	96	138	72	~ 30	11.1	4	1,3 [6].

Table S2: Description of B-stroke occurrence in response to sonar. TagID and description of the controlled exposure dives. RL refers to received sound pressure level (SPL) in dB re 1 μPa.

Tag ID	Scenario
md07_245a	Sonar started during the bottom phase of a deep dive (RLs 80-141 dB re 1 μPa) and continued for 2 min during the ascent phase (RLs 140-147 dB re 1 μPa), where only one B-stroke was produced when RL was 145dB re 1 μPa.
zc10_272a	Sonar started during the bottom phase of a deep dive (RLs 85-94 dB re 1 μPa) and continued during the ascent phase (RLs 103-128 dB re 1 μPa), 3 min within the ascent with a RL of 125 dB re 1 μPa B-strokes present until the end of the sonar exposure and beyond, until the animal reaches the surface. Some B-strokes were also present during the following shallow dive.
zc11_267a	Sonar occurred during most part of the descent phase of a deep dive (RLs 89-141 dB re 1 μPa), 6 min within the descent with a RL of 129 dB re 1 μPa B-strokes start and continue during the whole descent and ascent phase. No bottom phase present in this deep dive.
zc13_210a	Sonar started during the ascent phase of a shallow dive (RLs 106-111 dB re 1 μPa). First ping RL 107 dB triggers 2 B-stroke, but was 5 min within the ascent that B-onset occurs with a RL of 110 dB re 1 μPa. Although sonar pings continued to be emitted through the whole following shallow dive, they only started to be recorded on the tag, 4 minutes within the dive (RLs 114-124 dB re 1 μPa). 7 min within the dive B-strokes start with a RL of 116dB re 1 μPa. Sonar pings continued during the descent and bottom phase of the following deep dive. During this deep dive the tag was partially attached and falls off during the bottom phase, as such this deep dive was not included in the analysis.
ha13_176a	Sonar starts during the ascent phase of a shallow dive with only two pings (RLs 96-99 dB re 1 μPa), no B-strokes present. Sonar pings continue during the surface time and then during the descent phase of a deep dive (RLs 98-151 dB re 1 μPa), 8 min within the descent B-strokes start with a RL 138 dB re 1 μPa.
ha15_171a	Sonar started during the ascent phase of a short deep dive (RLs 66-85 dB re 1 μPa), no B-strokes present. Sonar pings continued during the whole following shallow dive and the descent of the next shallow dive (RLs 77-99 dB re 1 μPa), no B-strokes were present.
ha15_179b	Sonar started 2 min within the descent phase of a deep dive (RLs 114-127 dB re 1 μPa). First ping RL 127 dB re 1 μPa triggers 1 B-stroke but was 5 min within the dive descent that B-onset occurs with a RL of 125 dB re 1 μPa.
ha16_170a	Sonar started during the descent phase of a shallow dive (RLs 75-77 dB re 1 μPa), no B-strokes present. Sonar pings continued during the bottom phase (RLs 77-120 dB re 1 μPa) and ascent phase (RLs 116-124 dB re 1 μPa), 2.5 min within the ascent phase B-strokes start with a RL of 117 dB re 1 μPa. Sonar pings continue for less than 3 min during descent of the following shallow dive (RLs 117-121 dB re 1 μPa), no B-strokes present.
bb12_214a	Sonar started during the bottom phase of a shallow dive (RLs 101-133 dB re 1 μPa) and continued during the ascent phase (RLs 105-138 dB re 1 μPa), 1 minute within the ascent phase B-strokes start with a RL of 125 dB re 1 μPa. Sonar pings continued during the following shallow dive (RLs 106-113 dB re 1 μPa), 2 min within the dive (already during the ascent phase) B-strokes started with a RL of 111 dB re 1 μPa. Sonar pings continued for 3 min during descent of the following deep dive (RLs 108-112 dB re 1 μPa), B-strokes present at the first ping with a RL of 108 dB re 1 μPa.

Table S3. **Model selection B-stroke presence/absence at the dive level.** Model selection was carried out in two steps: among candidate models fitted to pre-exposure baseline data, and then fitting the best baseline model with two alternative sonar covariates to both pre-exposure and sonar exposure data. ΔAIC is shown with respect to selected baseline model (in bold) the lowest-AIC model in each dataset. Smooth covariates are denoted by s(). Abbreviations for the model structure stand as: dive_dur (*dive duration*), prev_dive_dur (*previous dive duration*) prev_Bn (*number of B-strokes in previous dive*), SA_rate (*regular fluke stroke rate during both descents and ascents*), SAdes_rate (*regular fluke stroke rate during descents*), sonar (*presence-absence of sonar*), SELcum (*cumulative SEL at the end of the dive*).

**Model**	**Dataset**	**Model_structure**	**ΔAIC**
1	Pre-exposure baseline	(intercept-only)	460.9
2		dive_dur	10.5
3		prev_dive_dur	453.1
4		prev_Bn	460.7
5		SA_rate	434.3
6		SAdes_rate	343.8
7		dive_dur + prev_dive_dur	7.0
8		dive_dur + prev_Bn	7.4
9		dive_dur + SA_rate	9.9
10		dive_dur + SAdes_rate	2.2
11		prev_dive_dur + SA_rate	424.0
12		prev_dive_dur + SAdes_rate	335.8
13		prev_Bn + SA_rate	431.7
14		prev_Bn + SAdes_rate	341.2
15		dive_dur + prev_dive_dur + SA_rate	6.9
**16**		**dive_dur + prev_dive_dur + SAdes_rate**	**0.0**
17		dive_dur + prev_Bn + SA_rate	7.5
18		dive_dur + prev_Bn + SAdes_rate	0.7
19		s(dive_dur) + s(SA_rate+ s(prev_dive_dur	18.0
20		s(dive_dur) + s(SAdes_rate) + s(prev_dive_dur)	12.2
21		s(dive_dur) + s(SA_rate) + s(prev_Bn)	18.3
22		s(dive_dur) + s(SAdes_rate) + s(prev_Bn)	12.6
**23**	Pre-exposure baseline + CEE	**dive_dur + prev_dive_dur + SAdes_rate**	**0.0**
24		dive_dur + prev_dive_dur + SAdes_rate + sonar	-15.7
25		dive_dur + prev_dive_dur + SAdes_rate + s(SELcum)	-17.5
26		dive_dur + SAdes_rate	1.9
27		dive_dur + SAdes_rate + sonar	-15.0
28		dive_dur + SAdes_rate + s(SELcum)	-17.2

Table S4: Model selection B-stroke proportion at the ping to ping level. The WAIC values are indicative of the model's predictive accuracy, with lower values representing a better balance between model fit and complexity. The models incorporate various predictors, including a fixed intercept (B_0), random effects based on each animal ($TagID$), nonlinear effects of sound pressure level (SPL), temporal correlation (Cor_{ar1}), and categorical variables for dive type and phase of the dive, including an interaction term between the latter two in model M4.

Model	Linear predictor	WAIC
Null	$B_0 + TagID + Cor_{ar1}$	**-3481.9**
**M0**	$B_0 + TagID + SPL + Cor_{ar1}$	**-3481.6**
M1	$B_0 + TagID + SPL + Cor_{ar1} + Divetype$	-3448.8
M2	$B_0 + TagID + SPL + Cor_{ar1} + Divephase$	-3477.3
M3	$B_0 + TagID + SPL + Cor_{ar1} + Divetype + Divephase$	-3480.8
M4	$B_0 + TagID + SPL + Cor_{ar1} + Divetype * Divephase$	-3478.7

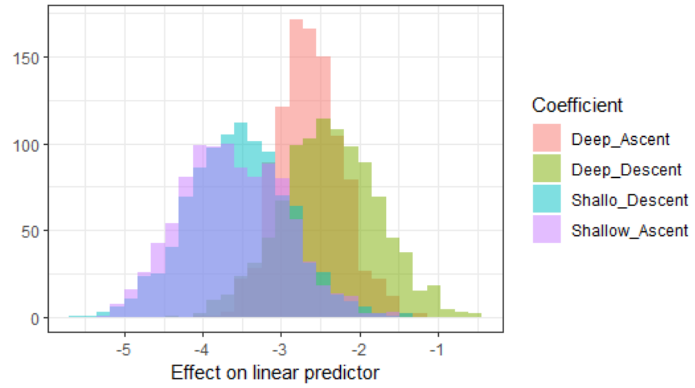

Figure S1: Ping-to-ping B-stroke proportion analysis Posterior distributions of the interaction coefficients between dive phase and dive duration (M4 model from **Table 4**). Note the similar distribution of dive phases within a dive type, but also the overlap between dive types meaning that there is no significant differences with the current data (which includes ping-to-ping data and also baseline data).

References

1. DeRuiter, S.L. et al. First direct measurements of behavioural responses by Cuvier's beaked whales to mid-frequency active sonar. *Biol. Lett.* **9**, 20130223 (2013).

2. Department of the Navy (DoN). Technical report: criteria and thresholds for U.S. navy acoustic and explosive effects analysis (Phase III). (SSC Pacific, San Diego, California, 2017).

3. Miller, P.J.O. et al. First indications that northern bottlenose whales are sensitive to behavioural disturbance from anthropogenic noise. *R. Soc. Open Sci.* **2**, 140484 (2015).

4. Stimpert, A.K. et al. Acoustic and foraging behavior of a Baird's beaked whale, *Berardius bairdii*, exposed to simulated sonar. *Sci. Rep.* **4**, 7031 (2014).

5. Tyack, P.L. et al. Beaked whales respond to simulated and actual navy sonar. *PLoS One* **6**, e17009 (2011).

6. Wensveen, P.J. et al. Northern bottlenose whales in a pristine environment respond strongly to close and distant navy sonar signals. *Proc. R. Soc. B* **286**, 20182592 (2019).
